# Editorial: Seeing is believing: cutting-edge technologies transforming ophthalmology

**DOI:** 10.3389/fmed.2025.1569161

**Published:** 2025-03-11

**Authors:** Miguel Angel Ariza-Gracia, Vijayavenkataraman Sanjairaj, Philippe Büchler

**Affiliations:** ^1^ARTORG Center for Biomedical Engineering Research, Faculty of Medicine, University of Bern, Bern, Switzerland; ^2^The Vijay Lab, Division of Engineering, New York University Abu Dhabi, Abu Dhabi, United Arab Emirates

**Keywords:** ocular biomechanics, finite element analysis, corneal crosslinking (CXL), 3D visualization, diabetic retinopathy surgery, keratophakia, femtosecond laser, artificial intelligence (AI)

## Introduction

Ophthalmology is entering a transformative era where innovation and clinical practice converge to revolutionize the diagnosis, management, and treatment of eye diseases. With millions worldwide relying on improved vision for a better quality of life, the significance of advancements in this field cannot be overstated. In this Research Topic of our journal, *Seeing is believing: cutting-edge technologies transforming ophthalmology*, we feature four studies that highlight the remarkable impact of emerging technologies on eye care. These articles cover innovations in corneal therapies, retinal surgery, refractive techniques, and artificial intelligence (AI), demonstrating how these innovations are reshaping the specialty and setting new standards for patient care.

## A new era for corneal cross-linking: precision at the microscopic level

The introduction of corneal cross-linking (CXL) for treating keratoconus has already proved revolutionary, with potential applications extending to non-invasive treatment of other refractive conditions such as myopia and hyperopia. However, as demonstrated in the study by Frigelli et al., the full potential of this therapy is only beginning to be realized. Utilizing optical coherence elastography (OCE), the study offers new insights into the biomechanical changes in the cornea during treatment and how the refractive changes are related to the biomechanical stiffening of the cornea.

The ability of OCE to measure axial elongation and stiffness in treated corneal regions marks a significant step toward personalized medicine. The study highlights that tailoring energy delivery during CXL and modifying irradiation patterns can enable patient-specific refractive corrections. For example, it was found that CXL treatment with high energy delivery can lead to a 2.4-fold increase in refractive correction compared to standard treatments. These findings open up the possibility of optimizing therapies for individual patients based on their unique corneal structures and needs.

Beyond immediate clinical applications, this study raises broader questions about corneal therapy. As our understanding of the interplay between corneal biomechanics and optics deepens, we can envision non-invasive, highly targeted solutions that not only halt the progression of keratoconus but also improve the optical performance of the cornea. However, achieving widespread clinical adoption, standardizing treatment protocols, and refining patient selection criteria remain challenges. This shift toward personalized refractive correction represents a paradigm shift in ophthalmology, placing the patient at the heart of innovation.

## Three-dimensional visualization in ophthalmology: faster, more precise proliferative diabetic retinopathy surgery

Minimally invasive vitrectomy poses a significant surgical challenge due to limited visibility of intraocular tissues, often necessitating repeated instrument use, prolonging operating time, and heightening the risk of complications such as retinal damage, bleeding, and detachment. While conventional microscope-based systems remain the gold standard, they are hindered by limitations in depth perception and ergonomics. In a study by Zhang et al., three-dimensional (3D) surgical video systems were compared to conventional microscopes, demonstrating the potential of advanced visualization technologies to overcome these challenges.

The study underscores the benefits of 3D systems in enhancing surgical precision and efficiency. By providing high-resolution stereoscopic views and better spatial orientation, 3D systems reduce surgical time and improve outcomes, as demonstrated by the shorter membrane removal time in the 3D systems group. Additionally, these systems alleviate surgeon fatigue through ergonomic improvements, making complex procedures less taxing and increasing the sustainability of high-volume surgical workloads.

The implications of 3D systems extend beyond Proliferative Diabetic Retinopathy (PDR) surgeries. As adoption grows, their potential to improve outcomes in cataract surgery, macular hole repair, and other vitreoretinal procedures is expected to become increasingly apparent. Furthermore, these systems hold immense potential as surgical training tools, offering trainees unparalleled clarity and detail compared to traditional systems. Despite these advantages, challenges such as the high cost of implementation and the learning curve for surgeons associated with transitioning from traditional microscopy remain. Nonetheless, the integration of 3D technology marks a significant leap forward in modernizing ophthalmic surgery and improving patient outcomes.

## On-demand keratophakia: e-beam storage and femtosecond laser

Advances in laser technology have played a critical role in refractive and corneal surgery, and the study by Chan et al. goes one step further by evaluating femtosecond laser systems in the context of electron-beam irradiated corneas. The research compares high-energy VisuMax lasers with low-energy FEMTO LDV lasers, analyzing their impact on lenticule quality and surface smoothness—critical factors influencing postoperative recovery.

The study shows that low-energy femtosecond lasers, such as the FEMTO LDV, produce smoother cut surfaces and cause less disruption to collagen morphology than high-energy systems. These findings are significant because smoother surfaces directly correlate with faster visual recovery and reduced post-operative complications. Additionally, the use of electron-beam irradiated corneas, which can be stored at room temperature for extended periods, addresses longstanding logistical challenges in corneal storage and transplantation.

The implications for stromal keratophakia and refractive surgery are profound. By ensuring better tissue quality and minimizing surgical trauma, these advances could lead to more predictable outcomes and broaden access to vision-restoring procedures. However, further evaluation of scalability and cost-effectiveness is necessary to facilitate widespread clinical adoption.

## Artificial intelligence: a revolution in pediatric ophthalmology

Early detection and treatment of amblyopia and its risk factors, such as strabismus and refractive error, are crucial to prevent long-term vision loss. However, conventional methods, including stereovision tests, often fall short in terms of sensitivity and scalability. The work of Csizek et al. introduces an AI-based solution that not only addresses these challenges but also sets a new standard for vision screening.

Their AI-based model integrates several non-stereoacuity-based tests, achieving higher sensitivity and specificity compared to classical stereovision tests such as Lang II and TNO. Particularly noteworthy is the study's use of an AI algorithm to synthesize test results, leveraging the strengths of individual assessments while compensating for their limitations.

An AI-driven, cost-effective screening tool could make early vision screening accessible even in resource-constrained environments. By reducing dependence on highly trained specialists, such solutions democratize healthcare and ensure that more children receive timely diagnoses and interventions. However, concerns about algorithm transparency, data privacy, and regulatory approval must be addressed to facilitate widespread adoption. As AI technology continues to evolve, its applications in detecting other ophthalmic conditions, from glaucoma to diabetic retinopathy, are likely to expand, solidifying its place as an indispensable tool in modern ophthalmology.

## Bridging technology and patient care: a vision for the future

The studies presented in this Research Topic exemplify the transformative potential of technology in ophthalmology. They illustrate a field where innovation is not an end but a means to improve patient care. From the precision of optical coherence elastography in corneal cross-linking to the immersive clarity offered by 3D surgical systems, and from the meticulous craftsmanship of femtosecond lasers to the diagnostic prowess of artificial intelligence, each advancement represents a significant step toward a future where visual disorders are not merely managed but anticipated and resolved with unparalleled precision.

